# C-854-05. From Chaos to Confidence: High-Fidelity Simulation for Burn Fluid Resuscitation Training

**DOI:** 10.1093/jbcr/irag033.081

**Published:** 2026-04-06

**Authors:** Brianna L Moorehead

**Affiliations:** UCHealth, Aurora, CO

## Abstract

**Introduction:**

Admitting a patient who requires fluid resuscitation can be a highly stressful and chaotic experience, particularly for healthcare providers with limited exposure to such critical scenarios. To prepare primary nurses for this complex process, initial training includes a didactic course featuring lectures, case studies, and hands-on simulation. Ongoing annual competency reinforcement is offered through diverse modalities such as podcasts, virtual training modules, and chart audits to support continued learning and confidence. This study explores the effectiveness of utilizing a burn admission simulation as an educational platform, specifically designed to engage kinesthetic adult learners in a safe and controlled environment.

**Methods:**

A high-fidelity simulation was designed for primary admitting nurses to practice assessments and workflows in the hydrotherapy room. Before starting, participants complete a pre-simulation knowledge assessment covering confidence in burn admission, delegation, and TBSA calculation. The simulation begins with the ICU nurse receiving report and entering the hydrotherapy room, where a moulaged mannequin is positioned. The nurse conducts a primary survey addressing deviations related to inhalation injury, respiratory effort, and full-thickness circumferential burns, followed by a secondary survey. Participants then calculate fluid rates, colloid volume, minimum rate, and IVY score. The session ends with a review of hourly documentation and additional care considerations related to mechanism of injury.

**Results:**

Over the course of a year, 5 Burn ICU nurses participated in the burn admission requiring fluid resuscitation simulation. Overall, the nurses’ confidence increased by 34% from “somewhat comfortable” to “very comfortable.” A 33% increase in knowledge related to calculating TBSA percentage was observed. Finally, a 34% increase was observed in proportion of participants who began the simulation as “somewhat comfortable” delegating tasks to “very comfortable.” Additional feedback from participants following the simulation included an appreciation for the “safe space to ask ‘what if’ questions in a chaotic, yet controlled environment.”

**Conclusions:**

The implementation of a high-fidelity burn admission simulation significantly improved nursing confidence and competence in key areas of burn care. Measurable increases in comfort with fluid resuscitation, TBSA calculation, and task delegation highlight the value of experiential learning in preparing nurses for complex clinical scenarios. Participant feedback further supports the simulation’s effectiveness in fostering a safe, supportive environment for skill development and critical-thinking.

**Applicability of Research to Practice:**

Any burn center focused on education for adult learners.

**Funding for the Study:**

N/A.

